# Plant-recycling of waste tyre rubber into asphalt binder for sustainability: Insight into physicochemical behavior during terminal production

**DOI:** 10.1371/journal.pone.0334132

**Published:** 2025-10-22

**Authors:** Hongwei Zhang, Xiaoxing Zhang, Haifeng Wang, Xiong Xu

**Affiliations:** 1 Yinchuan Branch of Ningxia Highway Management Center, Yinchuan, China; 2 School of Civil Engineering and Architecture, Wuhan Institute of Technology, Wuhan, China; 3 Hubei Provincial Engineering Research Center for Green Civil Engineering Materials and Structures, Wuhan Institute of Technology, Wuhan, China; Shandong University of Technology, CHINA

## Abstract

Crumb Rubber Modified Asphalt (CRMA) offers a vital pathway for global waste tire recycling, with swelling duration critically governing its performance during terminal production. This study examines physicochemical interactions between rubber particles and base asphalt under varied swelling durations. Systematic analyses employing extraction test, FTIR spectroscopy, rotational viscosity measurements, rheological test and Separation test assessed impacts on molecular structure, viscoelastic characteristics, rheological behavior and Storage stability. Results demonstrated that prolonged swelling time minimally influences residual rubber content (Δ ≤ 0.5%) but intensifies thermo-oxidative degradation of rubber particles in oxidation and desulfurization, thereby activating interfacial interactions of rubber particles and bitumen in CRMA binder. Extended swelling time will be able to initially elevate the viscosity through rubber-oil absorption and subsequently reduce the viscosity through dominant degradation, while progressively diminishing temperature susceptibility. Optimized swelling time at 4h can enhance complex modulus and viscoelastic balance through synergistic effects of rubber degradation and interfacial interactions, significantly improving high-temperature rutting resistance of CRMA binder. The softening point difference (SPD) of CRMA4 can be enhanced by 41% to satisfy better storage requirement. The study on the plant-recycling of waste tire rubber in modification of asphalt binder can provide more understandings in the rubberized asphalt binder production through combined analysis of rubber-asphalt compatibility and interfacial strength.

## Introduction

With the rapid development of the global automotive industry, the production and waste generation of tires have experienced explosive growth [1–3]. According to statistics, the global annual waste tires have exceeded 1 billion, and this number is still continuously increasing [4,5]. Traditional treatment methods (such as landfill or stacking) not only occupy a large amount of land resources but also cause serious environmental pollution problems, including soil degradation, groundwater pollution, and fire hazards [6–8]. At the same time, natural rubber, as a strategic resource, its global supply is becoming increasingly tight due to climate change and the reduction in planting area [9]. Behind this, it is of great importance to seek for a way to high-value consume discarded tire rubbers at large scale towards circular economy.

As of now, there are many different emerging technologies for the resource utilization of discarded tires, among which the technology stands out approaching to convert these waste tire rubbers into asphalt modifiers due to its economic efficiency and technical feasibility [10–12]. Previous studies have shown that the application of rubberized asphalt binder can not only effectively alleviate the environmental pressure caused by discarded tires but also significantly improve the engineering performance of asphalt pavements [13,14]. In the viewpoint of practical applications, it is known that rubberized asphalt pavement exhibits more excellent engineering performances, such as high-temperature permanent deformation resistance, low-temperature crack resistance, as well as fatigue resistance, which thus can benefit to extend the service life of asphalt pavement [15–17]. In addition, rubberized asphalt pavements also have the advantages of high driving comfort, excellent anti-skid performance, and strong water resistance, and have been widely used in highways and airport runways in many countries in Europe and America [18,19]. However, the overall performance of rubberized asphalt binder is highly dependent upon the compatibility between rubber particles and asphalt. Therefore, it is of great necessity to explore how to reach an enhanced rubber-asphalt compatibility while extending the swelling time and temperature, especially in plant-production [20,21].

Recent studies have shown that the interaction between rubber and asphalt during their high-temperature blending process mainly consists of the following two stages: the rubber particles physically swell and absorb the light components of the asphalt, causing volume expansion and forming a three-dimensional network; under the coupling action of high temperature and shearing, the rubber particles undergo desulfurization and chain-breaking reactions, being degraded into small molecular fragments and partially integrating into the asphalt matrix [22,23]. For instance, Xing et al. [24] investigated the rheological and swelling-degradation behaviors of rubber powder in asphalt matrices under varying swelling durations. Their findings indicated that prolonged swelling duration reduces CRMA viscosity and complex modulus while enhancing the penetration capacity of rubber particles within the asphalt matrix. Dong et al. [25] simulated rubber swelling in asphalt using SBR sheets instead of waste rubber powder, revealing that the swelling process rapidly increases then plateaus with prolonged duration, followed by structural degradation of the rubber network upon further extension. These studies demonstrated that the swelling duration of rubber particles can be used as a pivotal parameter that governs final performances of CRMA binder.

Although laboratory studies have already revealed the interaction mechanism of rubberized asphalt binder, there are significant differences between large-scale industrial production and laboratory simulations [26]. Specifically, the mixing equipment used in industrial production has a large volume and low heat transfer efficiency, which may lead to uneven swelling reactions [27]. Additionally, industrial production typically employs continuous stirring instead of the intermittent shear used in laboratories, and the shear rate and temperature field distribution are more complex [28]. Currently, research on the impact of swelling time on the performance of rubber-modified asphalt is still limited to the laboratory scale, and the interaction between rubber particles and asphalt binder during industrial production, as well as the influence of processing parameters on the performance of large-scale prepared rubber-modified asphalt during production, have not been deeply explored. To this end, it is necessary to fill the abovementioned research gap to extend the understanding of rubberized asphalt binder during the production in plant.

**Fig 1:**
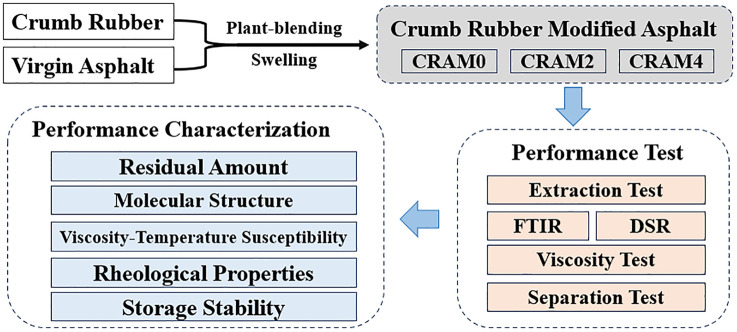
Flow chart of the research program.

On this basis, this study takes the rubber modified asphalt actually produced by a certain asphalt factory as the research object, and through tests such as residual rubber powder content, Fourier Transform Infrared Spectroscopy (FTIR), rotational viscosity test, Dynamic Shear Rheological (DSR) test, and storage stability test, comprehensively characterizes the microstructure, temperature-dependent properties, rheological properties, and storage stability of the modified asphalt. The aim is to explore the influence of swelling time on the performance in the process parameters of factory-scale rubber modification asphalt production, provide reliable data support for the industrial application of rubber modified asphalt, and promote the sustainable development of waste tire resource utilization technology. The flowchart of the research program is shown in Fig 1.

## Materials and methods

### Materials

**Fig 2:**
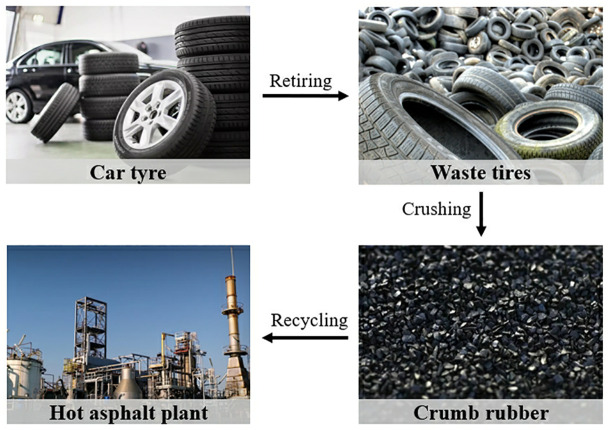
Recycling process of this factory.

**Rubber.** The discarded rubbers used in this study were mainly sourced from recycled truck and car tires, where the CR particles were originated from a local rubber recycling factory in Yinchuan, Ningxia province, China. This factory was equipped with a suit of crushing and grinding machines for powdering the rubbers. The brief description on the recycling of CR to asphalt binder modifications is displayed in Fig 2. In addition, the physical and chemical properties of rubber are presented in Table 1.

**Table 1:** Physical properties of crumb rubber.

Item	Measured value
Rubber size (mesh)	30 ~ 50
Carbon black content (%)	31 ~ 35
Ash content (%)	7.21
Relative density	1.15

**Virgin asphalt binder.** The virgin asphalt binder used was Pen.70 grade asphalt, provided by a local company. The physical properties of the virgin asphalt are shown in Table 2.

**Table 2:** Physical properties of virgin asphalt.

Item	Test result	Requirement	Standard
Ductility at 15°C (cm)	≥100	100	ASTM D113
Softening point (°C)	48.6	≥46	ASTM D36
Viscosity at 135°C (Pa s)	0.46	≤3	ASTM D4402
Penetration at 25°C (0.1 mm)	71	60-80	ASTM D5

**Fig 3:**
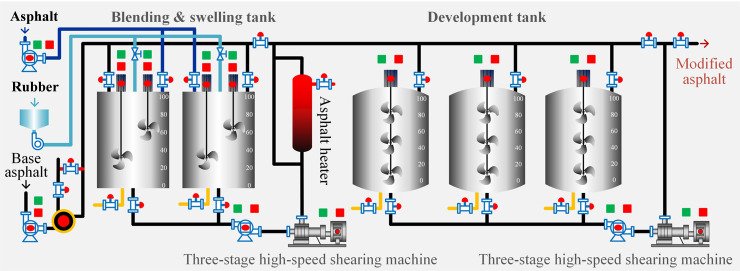
Production process of CRMA at the factory.

**Crumb rubber modified asphalt**. The crumb rubber modified asphalt (CRMA) was collected from the local asphalt binder production factory in Yinchuan, Ningxia province, China. During the production, the CR was added at 10%, by weight of virgin binder. The plant preparation of CRMA followed: first of all, the CR was weighed and added to molten virgin asphalt binder for mixing at 170 °C for 1 h, denoted as CRMA0; and then, the mixing condition was changed to 183°C for 2 h and 4 h to prepare CRMA binders, respectively, denoted as CRMA2 and CRMA4. To be clear, the main procedures are visually described and showed in Fig 3. After collecting, the physical properties of these CRMA binders were tested and shown in Table 3.

**Table 3:** Physical properties of the collected CRMA binders.

Items	Ductility at 5°C (cm, @ 1 cm/min)	Softening point (°C)	Penetration at 25°C (0.1 mm)
CRMA0	5.2	68.5	46
CRMA2	6.1	66.8	52
CRMA4	8.3	68.9	47

### Methods

#### Extraction test.

**Fig 4:**
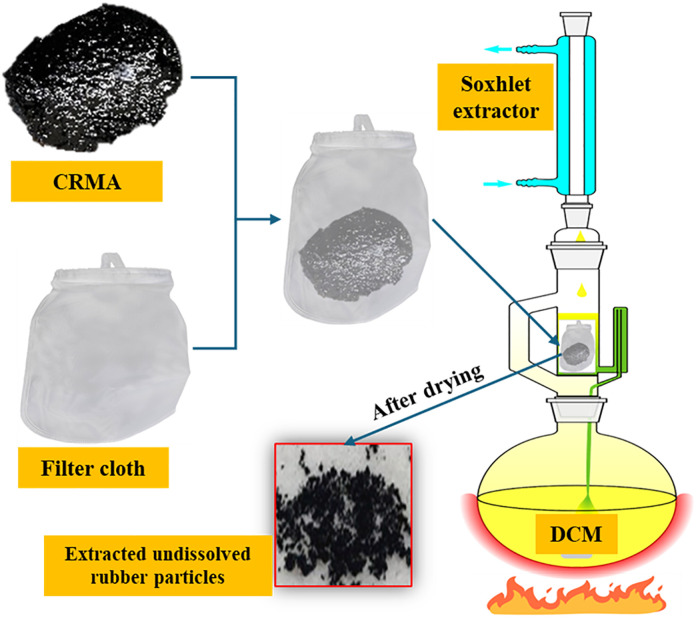
Extraction of CRMA binders to collect undissolved rubber particles.

**Fig 5:**
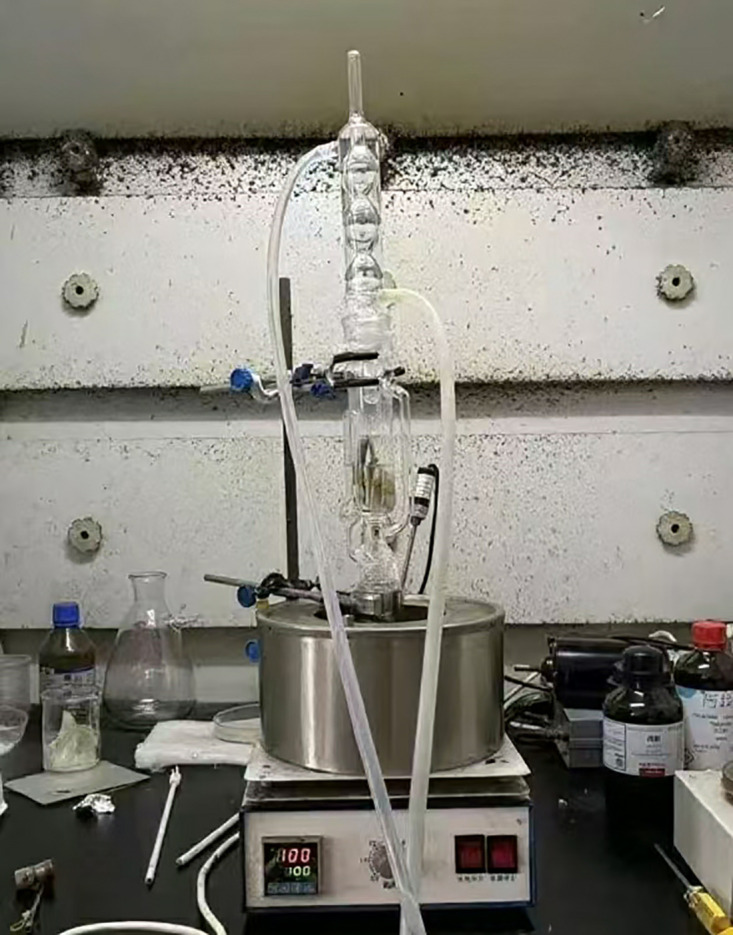
Extraction test device.

Residual rubber content in CRMA was precisely quantified using a Soxhlet extractor with dichloromethane solvent to directly evaluate the degradation extent of rubber particles during production. The procedure comprises dissolution, separation, and gravimetric steps. Specifically, CRMA samples were placed in Soxhlet extraction thimbles and subjected to exhaustive extraction with sufficient dichloromethane. Post-extraction, the solid residues (i.e., undegraded rubber) within the filter paper thimbles were rinsed with fresh dichloromethane, subsequently oven-dried, and cooled to ambient temperature. Finally, the residues were weighed on an analytical balance. The extraction of CRMA binders to collect undissolved rubber particles is shown in Fig 4. The extraction test device is shown in Fig 5.

The residual rubber content was calculated as shown in (1):


$$R=\frac{M_{\text{1}}}{M_{\text{0}}}\times \text{1}\text{0}\text{0}\%$$ (1)

where, *R* is the residual rubber content in CRMA, %; *M*_1_ is the mass of solid residues collected after extraction, g and *M*_0_ is the initial rubber mass in the original sample, g.

#### Fourier transform infrared (FTIR) spectroscopy.

The FTIR test was employed in this study to characterize the molecular structures of CRMA samples labeled as CRMA0, CRMA2, and CRMA4, with the aim of evaluating the effects of temperature and duration variations on their molecular configurations. A Nicolet Nexus 470 FTIR spectrometer was utilized to identify changes in functional group signatures at the molecular level. During testing, prepared CRMA samples were mounted on the sample holder’s scanning area, with instrumental parameters set as follows: wavenumber range of 4000–650 cm^-1^, 32 scans per measurement, and resolution of 4 cm^-1^. Data acquisition was initiated immediately after sample placement to minimize environmental interference.

#### Workability and viscosity-temperature susceptibility.

The rotational viscosity of CRMA binder was measured in this study using an NDJ-1F Brookfield viscometer at four temperatures (135°C, 150°C, 165°C, and 180°C) to characterize its workability during construction, where lower viscosity indicates better mixing efficiency. The testing strictly followed the T0625 standard in JTG E20-2011. No.27 spindle was selected for all tests, with torque maintained within 10%−100% of the instrument range. Each sample was tested in triplicate, and the average value was adopted as the final result to ensure repeatability (permissible error ≤3.5%). The viscosity-temperature susceptibility of CRMA was quantified by fitting the viscosity-temperature curve with the Saal formula (2):


$$\text{log}\;\text{log}\left(\eta \times {\text{1}\text{0}}^{\text{3}}\right)=n-m\;\text{log}(T+\text{2}\text{7}\text{3}.\text{1}\text{5})$$ (2)

where, η is the apparent viscosity of the asphalt binder, Pa s; T is the temperature, °C; n is the intercept of the fitted curve; and m is the slope of the fitted curve, which is positively correlated with the viscosity-temperature sensitivity of the asphalt binder.

#### Dynamic shear rheometer (DSR) test.

The rheological properties of CRMA binders were evaluated in this study using the MCR-502 dynamic shear rheometer (DSR) in accordance with AASHTO T315 specification. Temperature sweep test was conducted under controlled strain (1%) across a temperature range of 64–88°C with an interval of 6°C at angular frequency of 10 rad/s to characterize the viscoelastic behavior of CRMA under varying temperature and loading conditions. Based on this, the complex modulus (G*) and phase angle (δ) were obtained for analysis, and further the rutting factor (G*/sinδ) was calculated accordingly for evaluating the high-temperature features.

#### Separation test.

**Fig 6:**
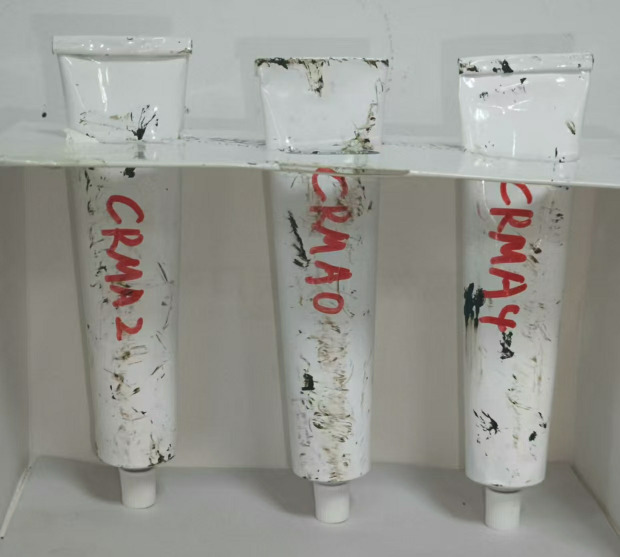
Separation test samples of CRMA.

The storage stability of CRMA was evaluated through a segregation test to assess the effect of swelling time on the compatibility between rubber and asphalt. According to ASTM D7173, the CRMA samples were poured into aluminum tubes (3.2 cm-diameter, 16 cm-height), sealed vertically, and placed in a high-temperature curing chamber at 163°C for 48 h. After curing, the samples were cooled to room temperature, and the tubes were sectioned into three equal parts. The softening point difference (SPD) between the top and bottom sections was determined, following ASTM D36, to quantify the storage stability of CRMA. The separation test samples of CRMA are shown in Fig 6.

#### Macroscopic appearance.

The surface smoothness of the rubberized binder increases with swelling time due to enhanced physicochemical interactions between crumb rubber and virgin asphalt. To qualitatively assess the rubber-bitumen interaction, the surface structure characteristics of the prepared samples were examined. High-resolution digital imaging was employed to capture detailed surface morphology for subsequent analysis.

## Results and discussion

### Residual amount of CRMA

**Fig 7:**
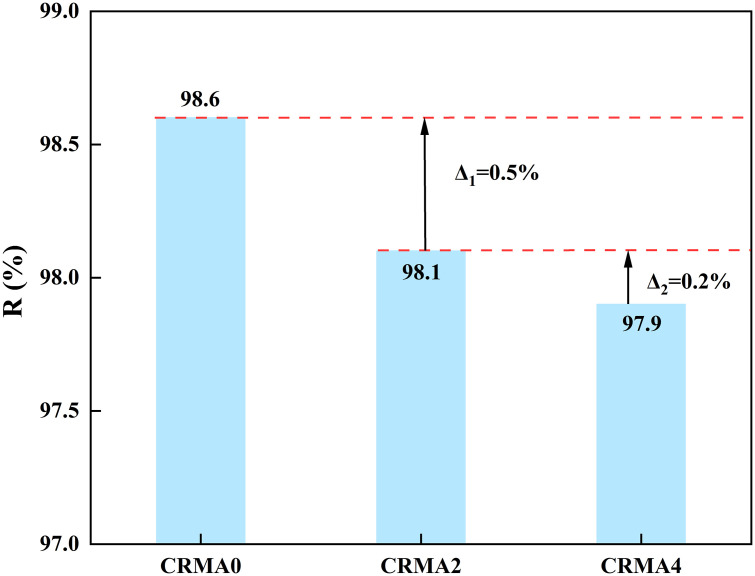
Influence of different swelling time on the residual amount of CRMA.

Fig 7 displays the residual rubber content test results for different CRMA samples. As shown in Fig 7, compared with CRMA0, the residual rubber content of CRMA2 decreased from 98.6% to 98.1% (Δ₁ = 0.5%). When the swelling duration increased from 2 to 4 h, CRMA4 exhibited a residual rubber content of 97.9% (Δ₂ = 0.2%). Under high-temperature conditions, the residual rubber content progressively decreased but with diminishing reduction rates. These results indicate that swelling duration had limited impact on residual rubber content. Additionally, CRMA0 showed residual rubber content below the theoretical added value (R < 1), possibly due to incomplete extraction.

### Molecular structure

**Fig 8:**
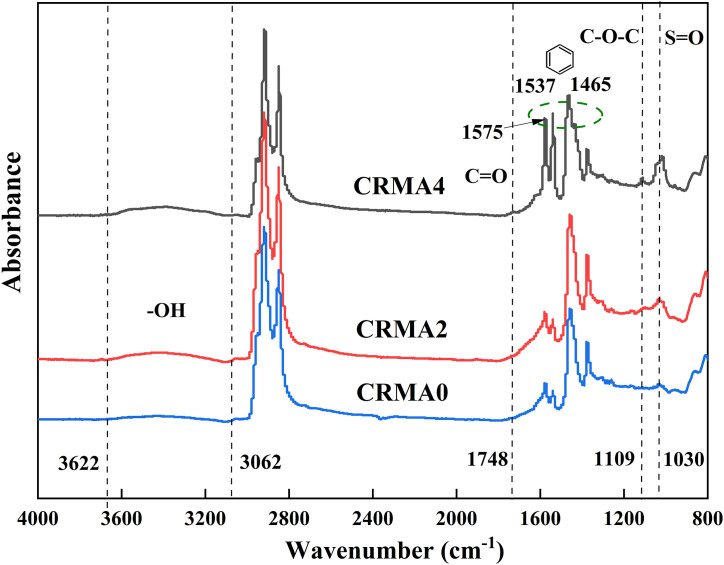
Influence of different swelling time on the molecular structure of CRMA.

Fig 8 reflects the molecular structural changes of CRMA binder under different swelling durations. In addition, the attribution of main characteristic bands of CRMA binders are analyzed and shown in Table 4. It is clear that no significant shift in characteristic absorption peaks occurs during the continuous swelling process of rubber-asphalt blending, confirming that the modification process is primarily physical blending with minor chemical reactions. Compared to CRMA0, as temperature increases and swelling duration extends, CRMA2 exhibits intensified absorption peaks at 1748 cm^-1^ (attributed to C = O carbonyl stretching vibration), 1109 cm^-1^ and 1030 cm^-1^ (both assigned to C-O-C stretching vibration), as well as at 1030 cm^-1^ (associated with S = O stretching vibration). These spectral changes clearly demonstrate the occurrence of oxidation reactions and sulfur bond cleavage during rubber-asphalt modification. With further extension of swelling time, CRMA4 shows reduced peak intensities at these positions, indicating that prolonged exposure to elevated temperatures progressively accelerates oxidation and desulfurization.

**Table 4:** Attribution of main characteristic bands of CRMA.

Wavenumber (cm^-1^)	Structure vibration stype
3623 ~ 3061	O-H stretching
1748	C = O stretching
1575,1537,1465	Benzene ring skeleton vibration
1109	C-O-C stretching
1030	S = O stretching, C-O-C stretching

The absorption peaks at 1575 cm^-1^, 1537 cm^-1^ and 1465 cm^-1^ (corresponding to benzene ring skeleton vibrations) exhibit noticeable intensity reduction after heating and sustained swelling. The absorption band between 3622 cm^-1^ and 3062 cm^-1^ (assigned to O-H stretching vibration) displays an initial increase followed by decrease in intensity, which may result from the physical swelling process promoting asphalt component penetration into rubber particles while simultaneously causing migration of rubber decomposition products into the asphalt matrix [29]. Subsequent desulfurization and degradation reactions consume hydroxyl groups in the asphalt. During swelling, the high-temperature environment facilitates oxidation of unsaturated double bonds in rubber by oxygen, generating carbonyl (C = O) compounds. Meanwhile, the original crosslinked sulfur bonds (S-S) in vulcanized rubber undergo thermal-oxidative cleavage and transform into sulfoxide groups (S = O). Under combined thermal, oxidative and mechanical stresses, rubber macromolecules experience main chain or side chain scissions, leading to structural damage, with the broken chains participating in new reactions with asphalt components [30].

### Workability and viscosity-temperature susceptibility

**Fig 9:**
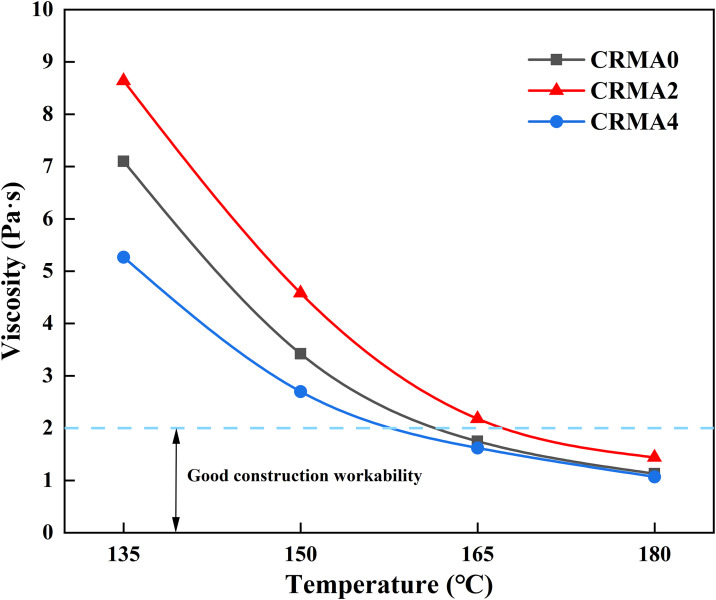
Influence of different swelling time on the viscosity of CRMA.

Fig 9 indicates the influence of swelling duration on the viscosity-temperature characteristic of CRMA binder. As displayed in Fig 9, the viscosity of CRMA binder continuously decreases with the increasing temperature. At each test temperature, the viscosity follows the order: CRMA2 > CRMA0 > CRMA4. These results demonstrate that the viscosity of rubberized asphalt undergoes a phased change during continuous swelling—initially increasing and then decreasing.

This phenomenon can be attributed to the following mechanisms: In the early swelling stage, rubber particles rapidly absorb light components (aromatics and saturates) from the asphalt, leading to volumetric expansion and the formation of a high-viscosity gel membrane. The interconnected gel membranes create a three-dimensional physical cross-linked network that restricts the free movement of asphalt molecular chains, resulting in a rapid viscosity increase. As swelling progresses, the rubber particles reach their maximum expansion capacity, and the sulfur crosslinks (S-S) begin to break, causing the degradation of the cross-linked network and a significant viscosity reduction [31]. Concurrently, the depletion of light components in the asphalt further diminishes viscosity. Notably, all CRMA samples exhibit viscosity values below 2.0 Pa· s at 180°C, indicating excellent workability for construction applications at this temperature.

**Fig 10:**
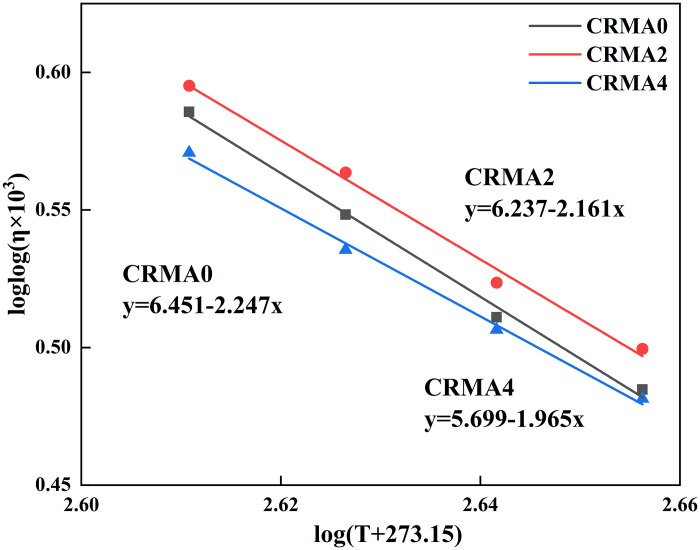
Influence of different swelling time on the temperature sensitivity of CRMA.

Fig 10 shows the fitted viscosity-temperature curves for analyzing the temperature-viscosity sensitivity of CRMA, with the regression equations and key parameters listed in Table 5. The correlation coefficients (R²) of all fitted curves exceeded 0.98, indicating the fitted curves accurately represent the relationship between viscosity and temperature. The fitting results revealed that the m-values for CRMA0, CRMA2, and CRMA4 were 2.247, 2.161, and 1.965, respectively, showing a consistent decreasing trend. This indicated that the prolonging of blending time gradually can reduce the temperature-viscosity sensitivity of CRMA.

**Table 5:** Saal equation and VTS of CRMA.

Items	Linear fitted equation, Saal form	m (VTS)	R2
CRMA0	loglog(η)=6.451-2.247log(T + 273.15)	2.247	0.993
CRMA2	loglog(η)=6.237-2.161log(T + 273.15)	2.161	0.989
CRMA4	loglog(η)=5.699-1.965log(T + 273.15)	1.965	0.994

During high-temperature blending, crumb rubber continuously absorbs light components (saturates and aromatics) from the asphalt, increasing the proportion of heavy components (asphaltenes) in the matrix. Since heavy components exhibit lower temperature sensitivity, this shift contributes to the overall reduction in viscosity-temperature dependence [32]. Additionally, extended blending promotes chemical bonding between rubber and asphalt, enhancing interfacial compatibility. Compared to physical entanglement, chemical bonds (sulfur bridges or oxidized functional groups) are more thermally stable, thereby reducing viscosity fluctuations caused by temperature variations.

### Rheological properties

#### Complex modulus.

**Fig 11:**
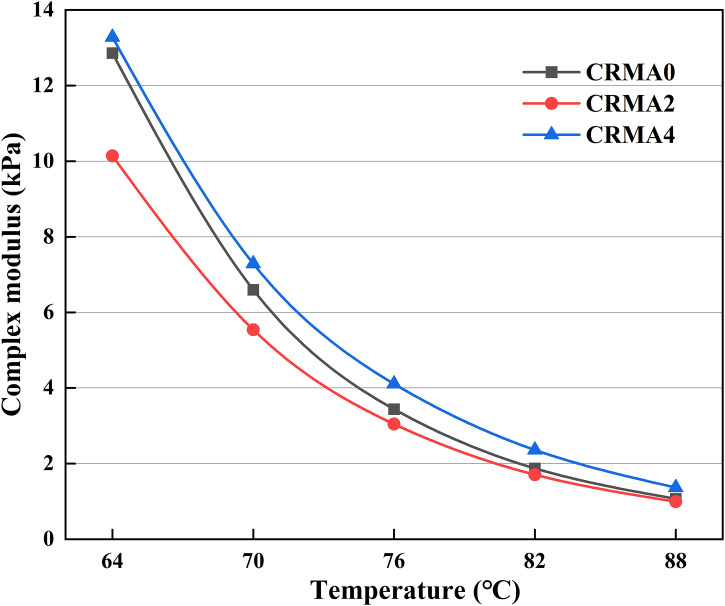
Influence of different swelling time on the complex modulus of CRMA.

Fig 11 shows the influence of different swelling time on the complex modulus of CRMA. The results indicate that within the temperature range of 64°C to 88°C, the complex modulus of all CRMA gradually decreases as temperature increases, reflecting a reduction in their deformation resistance. This occurs because asphalt exhibits elastic behavior at lower temperatures, providing stronger shear resistance [33]. As temperature rises, the asphalt transitions to a viscoelastic state, leading to diminished shear resistance. Compared to CRMA0, CRMA2 shows a decrease in complex modulus across all temperatures, with the decline becoming less pronounced at higher temperatures. However, when the swelling time is extended from 2h to 4h, CRMA4 demonstrates a significant increase in complex modulus, surpassing CRMA0 at every tested temperature. This suggests that a swelling duration below 2h under high temperatures reduces the modulus of CRMA, impairing its rutting resistance, whereas extending the swelling time to 4h markedly enhances this performance.

The observed trends can be attributed to the following mechanisms: during the initial 2h of high-temperature swelling, rubber particles partially absorb the light components of the asphalt, causing them to swell and soften. These swollen rubber particles act similarly to plasticizers or softeners within the asphalt matrix. Additionally, the absorption of light components by the rubber particles alters the local composition of the surrounding asphalt (enriching asphaltenes while depleting light fractions). These combined effects reduce the overall stiffness and shear resistance of the asphalt matrix, particularly at the starting test temperature of 64°C.

When the swelling time is prolonged to 4h, the rubber particles undergo more extensive swelling and degradation. Their particle size further decreases, increasing the specific surface area and enhancing the interfacial contact between rubber and asphalt. Moreover, prolonged swelling promotes stronger physical adsorption and oxidative chemical reactions between the rubber particles and asphalt, forming a tighter and more robust interface. These mechanisms significantly improve the rigidity of the modified system, enabling it to better resist shear deformation at high temperatures, ultimately resulting in the elevated complex modulus of CRMA4.

### Phase angle

**Fig 12:**
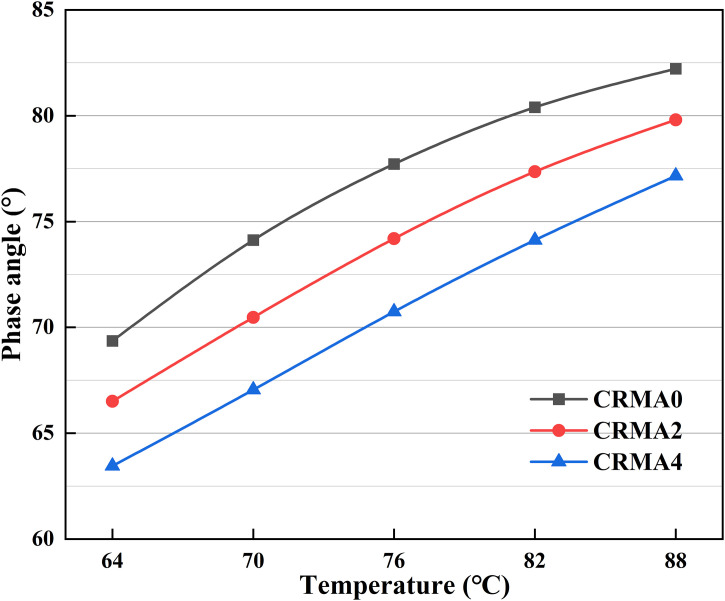
Influence of different swelling time on the phase angle of CRMA.

Fig 12 shows the influence of different swelling time on the phase angle of CRMA. As the test temperature increases from 64°C to 88°C, the phase angles of all CRMA samples rise, indicating enhanced viscous behavior and reduced elasticity at higher temperatures, consistent with the thermal sensitivity of asphalt binder [34]. Compared to CRMA0, CRMA2 (with 2h of high-temperature swelling) exhibits a significant decrease in phase angle. When the swelling time is extended to 4h, CRMA4 shows a further reduction in phase angle. At the same temperature, the phase angles follow the order: CRMA0 > CRMA2 > CRMA4, suggesting that CRMA4 has the lowest temperature sensitivity, while CRMA0 is dominated by viscous flow, making it more prone to deformation at high temperatures.

These results demonstrate that increasing swelling time reduces the phase angle of rubber-modified asphalt, significantly improving its elasticity and resistance to rutting. During the initial swelling stage, rubber particles retain their large size and intact structure, absorbing light components from the asphalt and forming weak interfacial layers that promote viscous flow. With prolonged swelling, the rubber undergoes sufficient degradation, increasing its specific surface area and enhancing chemical crosslinking with the asphalt, thereby improving the high-temperature stability of the modified asphalt.

### Rutting factor

**Fig 13:**
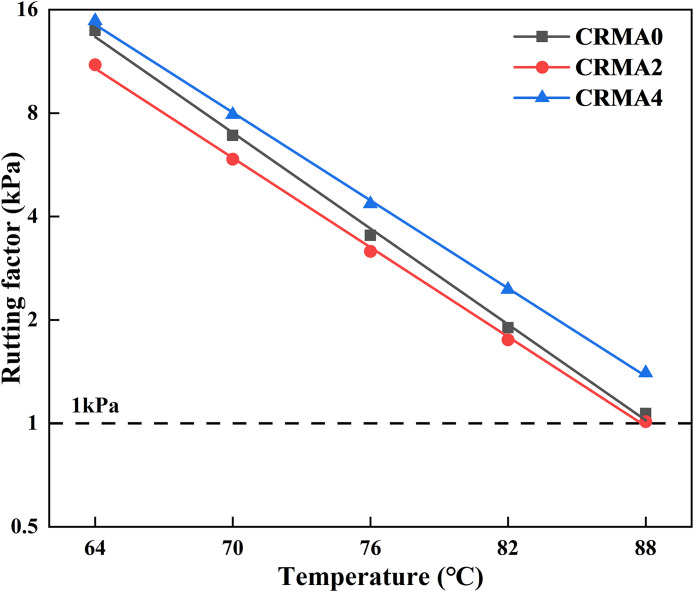
Influence of different swelling time on the rutting factor of CRMA.

Fig 13 shows the rutting factors of different CRMA, with their corresponding critical rutting temperatures and high-temperature grades listed in Table 5. As observed in Fig 13, the rutting factors of all CRMA samples decrease with increasing test temperature. Compared to CRMA0 (unswollen), CRMA2 (2h swelling) exhibits a reduced rutting factor, though the decline diminishes at higher temperatures. When swelling time extends to 4h, CRMA4 demonstrates a significantly higher rutting factor than CRMA0, indicating that optimal swelling duration (4h) enhances the asphalt’s resistance to shear flow and improves anti-rutting performance.

Table 6 reveals that the critical rutting temperatures shift from 88.2°C (CRMA0) to 88.0°C (CRMA2) and 90.6°C (CRMA4), while all samples maintain a high-temperature grade of PG82. During initial swelling (2h), rubber particles absorb light components from the asphalt, dispersing within the matrix and altering the local composition of surrounding asphalt. This reduces the overall stiffness and rutting resistance, leading to a lower rutting factor. Prolonged swelling (4h) promotes deeper degradation of rubber particles, increasing their specific surface area and fostering stronger interfacial bonding with the asphalt. The resulting rigid rubber-asphalt system significantly enhances the rutting factor.

**Table 6:** Critical rutting temperatures and high-temperature grades of CRMA.

Items	Critical rutting temperature	High-temperature PG grade
CRMA0	88.2	PG82
CRMA2	88.0	PG82
CRMA4	90.6	PG82

### Storage stability

**Fig 14:**
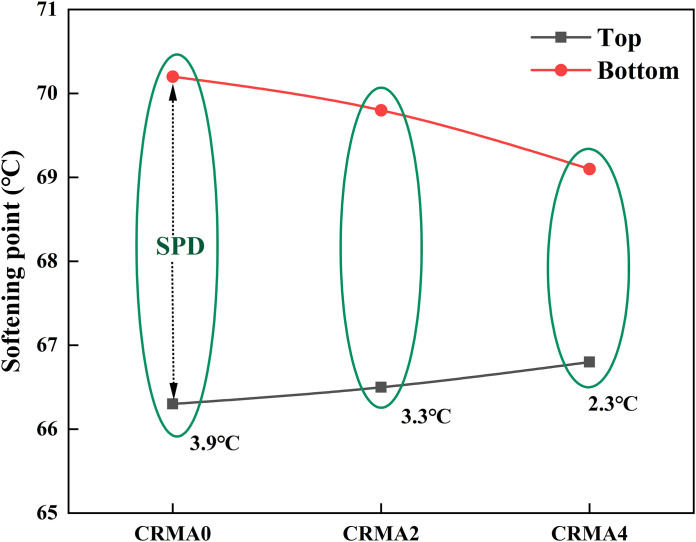
Influence of different swelling time on the storage stability of CRMA.

Fig 14 presents the influence of different swelling time on the storage stability of CRMA. As shown in Fig 14, the SPD (separation potential difference) value of CRMA gradually decreases with increasing swelling time. When the swelling time extends to 4h (CRMA4), the SPD reduces to 2.3°C, marking a 41% improvement compared to CRMA0 (SPD = 3.9°C). These results indicate that the compatibility between rubber particles and asphalt enhances with prolonged swelling time. During the initial swelling stage, larger rubber particles (with lower density than asphalt) tend to float, leading to rubber enrichment at the top and asphalt accumulation at the bottom. As swelling time increases, the rubber particles undergo oxidation and desulfurization, gradually expanding in volume and forming a crosslinked network with the asphalt. This network structure effectively suppresses phase separation in the modified asphalt system [35].

### Surface appearance

**Fig 15:**
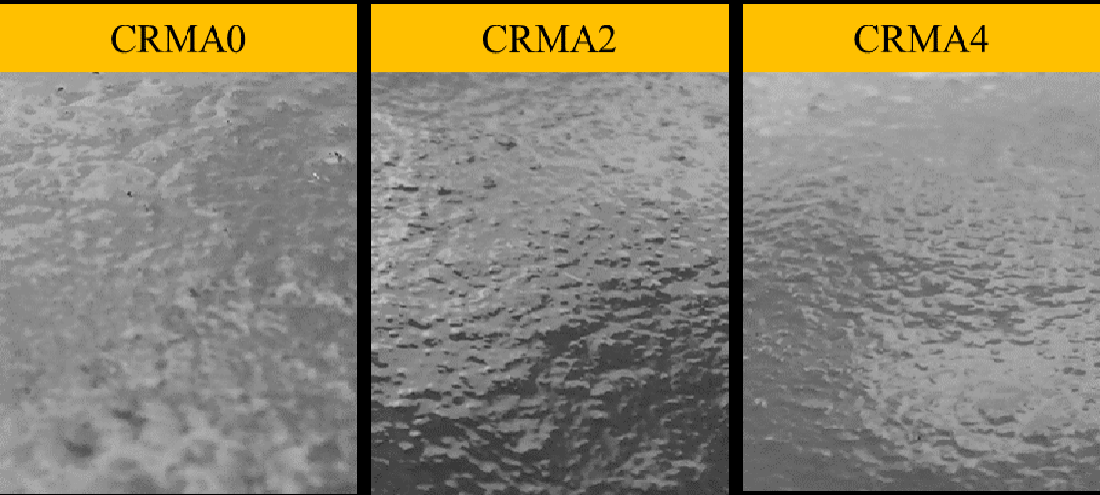
Surface appearances of plant-blended CRMA binders sampled at 0h, 2h, and 4h.

Fig 15 displays the surface appearances of plant-blended CRMA binders sampled at 0h, 2h, and 4h. It is clear that the rough surface of CRMA binder becomes gradually smoother with plant-blending time from 0h to 4h. Although the surface appearance of plant-blended CRMA binder presents to be relatively plain and smooth with time at elevated temperatures, the smaller rubber particles are still visible, showing the significant surface difference from the laboratory high-speed-shearing-mixed CRMA binders which own an almost particle-free surface [36]. These results indicated that particulate CR dispersed in asphalt binder happens with first swelling stage and second degradation stage at high temperatures during plant-blending production within limited allowed mixing time in consideration of cost-effectiveness.

## Conclusions

This study investigated the effects of swelling time on the performance of CRMA based on industrial production processes, utilizing extraction tests, FTIR spectroscopy, rotational viscosity testing, DSR test, and separation tests. On this basis, the following conclusions can be drawn:

- Extraction tests indicated a negligible change in residual crumb rubber content (Δ ≤ 0.5%) despite prolonged swelling from 2 h to 4 h.
- FTIR analysis demonstrated that prolonged swelling time accelerated oxidation and desulfurization via thermo-oxidative coupling, evidenced by carbonyl/sulfoxide group formation, transient hydroxyl consumption, and weakened aromatic structure.
- Workability and viscosity-temperature susceptibility tests revealed that viscosity of CRMA exhibited a biphasic trend-initially increasing then decreasing with prolonged swelling. Compared to CRMA0 and CRMA4, CRMA2 demonstrated superior workability and fluidity at identical temperatures. Furthermore, viscosity-temperature susceptibility progressively declined with extended swelling duration.
- DSR test showed that extending swelling time to 4 h significantly enhanced the high-temperature rutting resistance of rubber-modified asphalt through synergistic effects of thorough rubber particle.
- Separation test confirmed that optimal stability of CRMA was achieved after a 4h swelling process, with significantly enhanced compatibility between crumb rubber particles and the asphalt matrix.
- Surface appearance observation indicated that rubber particles can be well dispersed in asphalt binder with first-swelling-and-then-degradation behaviors at elevated temperatures.

Overall, this study investigated the plant-recycling of waste tyre rubber into asphalt binder for modification with swelling time, giving the new understanding on the real-world interactions between rubber and bitumen during production. Research limitation lies in lacking the evaluation regarding the controlling relationship between emission and quality of CRMA during hot-mix production, which is extremely important to the practical production in reality. Furthermore, the assessment of the benefits and costs of factory production also requires in-depth exploration. Future studies may need focus on comprehensive assessments of engineering and environmental performances of plant-blended CRMA for more sustainable applications.

## Supporting information

S1 File The original data file of the test. (XLSX)
